# A framework for the implementation of certification procedures in nurse level: a mixed approach study

**DOI:** 10.1186/s12913-021-06940-0

**Published:** 2021-09-08

**Authors:** Israa Salma, Mathias Waelli

**Affiliations:** 1 École des Hautes Etudes en Santé Publique, 7348 MOS Rennes, EA France; 2 École des Hautes Etudes en Santé Publique, 7348 MOS Paris, EA France; 3 Global Health Institute, Geneva University, Geneva, Switzerland

**Keywords:** Implementation, Certification, Local context, Nurse activities, Managers, Framework, Components

## Abstract

**Background:**

The implementation of certification procedures across healthcare systems is an essential component of the management process. Several promising approaches were developed toward a successful implementation of such policies; however, a precise adaptation and implementation to each local context was essential. Local activities must be considered in order to generate more pragmatic recommendations for managers. In this study, we built a framework for the implementation of certification procedures at nurse activity level. This was developed using two objectives: the identification of key implementation process components, and the integration of these components into a framework which considered the local socio-material context of nurses’ work.

**Methods:**

We used a two-step mixed approach. The first was inductive and consisted of a qualitative case study conducted between April and December 2019. Here, we analyzed the implementation of certification procedures in a French teaching hospital. Data were collected using semi-structured interviews and observations. In the second approach, emerging data were deductively analyzed using the Quality Implementation Tool (QIT) and Translational Mobilization Theory (TMT). Analyses were combined to construct an implementation framework.

**Results:**

Sixteen interviews were conducted with participants from different organizational levels, managers, mid-managers, and nurses. Additionally, 83 observational hours were carried out in two different wards. Our results showed that, (1) All retrieved elements during the process were successfully captured by the QIT components, only one component was not applicable. (2) We identified elements related to the local activity context, with the different interrelationships between actors, actions, and contexts using the TMT. (3) Our analyses were integrated and translated into a framework that presents the implementation of certification procedures in healthcare facilities, with a specific interest to the nurse/mid-manager level. By initially using QIT, the framework components took on a transversal aspect which were then adapted by TMT to the local work context.

**Conclusions:**

We successfully generated a framework that supports the implementation of certification procedures at the activity level. Our approach identified a broader vision of the interactions between proximity managers, teams, and contexts during change mobilization, which were not encompassed by transversal framework only, such as QIT. In the future, more empirical studies are needed to test this framework.

**Supplementary Information:**

The online version contains supplementary material available at 10.1186/s12913-021-06940-0.

## Background

Healthcare systems are becoming increasingly complex, where individual patients receive care from multiple providers and a multitude of professionals, within a context of reduced and regulated hospitalization procedures [1]. Considerable efforts have been made to improve the care quality and patient safety, as evidenced by the proliferation of checklists, protocols, and attempts to standardize care pathways [2]. Unequivocally, these factors impact professionals’ workloads, especially nursing groups [3], who are the largest providers of continuous patient care [4].

Quality measurement and management approaches play significant roles in reform; however, they constitute a timely consideration for healthcare managers and policy makers in terms of their preparation and implementation in professional daily practices [5]. Since 2004, quality certification has been a major external quality evaluation procedure in the French healthcare system [6]. It is iterative and mandatory for all public and private healthcare facilities and is conducted every four or six years [7]. This “peer evaluation technique” is based on the International Organization for Standardization (ISO) [8] which not only considers the quality and safety of care provision, but also continuously enhances an organization’s performance and improves patient satisfaction [9]. Certification has gradually evolved from promoting and integrating quality improvement initiatives [6, 10], to measuring implementation metrics in line with increased risk management and patient care [10]. The most recent certification process was synchronized with each establishment procedures, where it was based more on the quality monitoring tool, Compte Qualité (CQ), which reflected each institution’s commitment to quality and risk management systems and process improvement [10].

Certification evaluation strategies rely on standards and benchmarking and must therefore encompass best clinical practices and care process audits [6], and be well supported by quality and safety indicators (Indicateur de Qualite et Securite des Soins, IQSS) [11, 12]. Thus, the approach has implemented several care pathways, protocols, and checklist models to manage quality and reduce risk [2]. For example, quality and risk management items include - as outlined in the French National Health Authority (Haute Autorite de Sante, HAS) certification manual - a comprehensive criteria list comprising policies governing quality and care safety improvements, professional practice evaluation (Evaluation des Pratiques Profesionnels, EPP), document management, and adverse event management [13]. These high governance exigencies are both prominent and essential in high risk sectors to manage risk and control safety in terms of professional practice [14]. However, these requirements also generate large workloads for nurses [3] and are primarily due to the major roles nurses have in daily practice e.g., implementing and monitoring certification procedures. Nurses are familiar with management, leadership and auditing issues given their academic background [15]. Thus, certification procedures are major strategic and managerial issues for healthcare organizations in terms of preparation, implementation, and day-to-day sustainability [16].

In terms of implementation, the literature offers several promising approaches [17, 18] where key attributes, facilitators, and barriers come together to promote effective implementation strategies [18, 19] of this dynamic process [20]. In 2015, Nilsen et al. generated a differentiating approach incorporating three main aims [18]; a *process model* which described and guided the translation of research into practice [19, 21]; a *determinant framework* which explained and attempted to understand what influenced implementation outcomes [22–24], and *evaluation frameworks* which evaluated implementation efforts [25, 26]. These approaches generally emphasized systematic and cross sectional factors such as leadership, organizational culture, and the availability of time, materials and resources [27]. However, it is also important to define these transversal components at the activity level, to understand how interventions could become embedded into activity systems, and to identify implications for healthcare quality [28]. To this end, several recent studies have stressed the importance of local socio-material infrastructures, their effects on change integration [27], and how they are pivotal in generating quality improvement results [29]. However, there is a dearth of professional frameworks related to nurses’ activities in the literature, specifically nursing mandates in terms of essential roles, either directly in patient care and/or indirectly in coordinating activities and organizational care [2], and the plethora of practice requirements which come under quality assurance perspectives.

In this study, we constructed a framework for the implementation of certification procedures at the nurse activity level. This determinant framework seeks to facilitate implementation endeavors by presenting an extended vision from the generic factors impacting an implementation process to local socio-material factors such as local work dynamics. This was this was based on a mixed approach design covering two main objectives; firstly, we identified and framed key implementation components based on a qualitative study and the incorporation of a practical implementation science tool. Secondly, we integrated these components into a framework which considered specific local socio-material contexts. A socio-material context reflects both socio- and material elements which can be interwoven and constitute the local context of the activity, in our case nurse activities [27].

## Methodology

### Study design

**Fig. 1:**
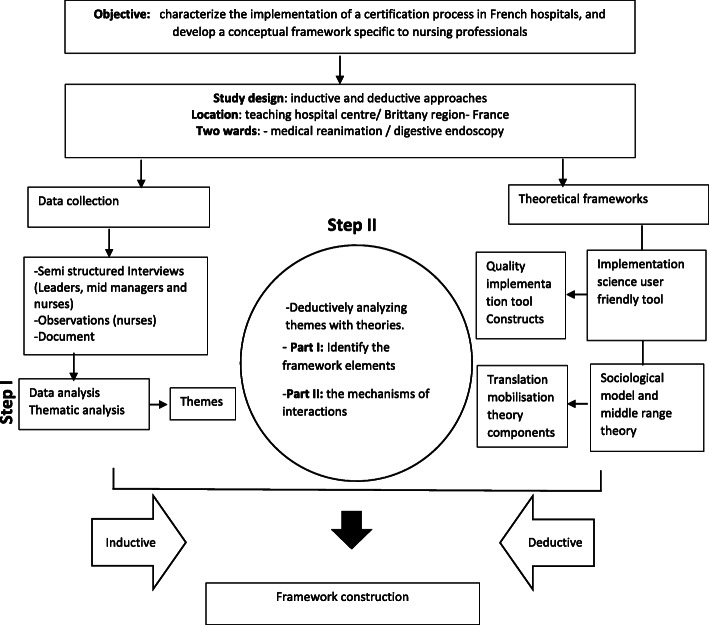
A flow diagram summarizing study design and output. (adapted from Creswell and Plano Clark [33])

This study was conducted based on a mixed two-step approach (Fig. 1). The first inductive step was a qualitative case study which allows researchers to investigate phenomena in natural or ‘real life’ contexts [30], examine closely how events occur, and understand the implementation of interventions in the healthcare systems [31, 32]. In a second step, the emergent themes were deductively analyzed using two different theoretical approaches; a practical implementation science tool and a middle range theory. This triangulation process between the different approaches provided the basis for a framework. At the final stage, the combination of results led to the construction of framework.

### Study location

This study was performed between April and December 2019 in a large teaching hospital (924 beds) in western France. The hospital previously passed four certification processes and was awarded a B rank without recommendations during the last visit. Data were collected from two high risk wards: medical reanimation (Med Rea) and digestive endoscopy (Dig Endo). These wards required a high governance status in terms of patient care and nurse practices as identified in the certification manual. These wards were therefore ideal locations to conduct our study.

The Med Rea ward has a patient/nurse ratio of 5:2. Here, seriously ill patients required respiratory assistance and were dependent on nursing and medical care. Med Rea nurses were qualified to manage and respond to contingencies and unexpected situations. The electronic health record (EHR) system in this ward was partially integrated, therefore a combination of electronic and paper records were used.

The Dig Endo ward functioned under a predefined intervention schedule; on average it experienced eight programmed interventions/day/room over a 10 h shift, five days/week. The area was highly technical, with a high patient rotation and an integrated EHR system.

### Data collection

Data came from semi-structured interviews and observations and were supported by documents relevant to certification procedures.

### Interviews

Interviews were conducted with actors from different hierarchical levels involved in the implementation of certification procedures, e.g., leader, mid-manager, and nurse levels. This strategy provides an in-depth insight into their experiences, perspectives, and roles. It also captures the issue from multiple lenses allowing a better understanding multiple facets for certification procedures implementation processes [34]. Sampling of interviews was performed based on a data-saturation approach, which means that the interviews’ output reached a sense of closure because the new interviews yielded non-essential information in terms of study aims [35]. The semi-structured interviews were conducted by the principle investigator (PI) only. The interview guide was covering the following topics: quality approaches in the hospital, certification procedures and implementation processes for certification procedures, key factors, barriers and facilitators and their impact on nurse activities.

After the initial e-mail contact and the obtainment of written informed consents to participate provided by the participants, the primary phase interviews commenced with nursing leaders and managers. Nurse interviews were conducted during the observations on wards. Nurses with at least one year of work experience and having the French national diploma in nursing science were selected as basic qualification levels, to avoid knowledge or experience bias in the sector. Nurse demographic characteristics are shown (Table 1). All interviews were recorded and transcribed.

**Table 1:** Participant demographics (for the eight participating nurses)

Participant demographics		Med Rea	Dig Endo
Age (years)	30–45	4	2
	> 45	0	2
Gender	female	4	3
	male	0	1
Work experience (years)	< 10	0	2
	> 10	4	2
Experience in ward (years)	1–5	2	2
	> 5	2	2
Education	RN^a^	3	4
	HD^b^	1	0

^a^*RN* Registered Nurse; ^b^*HD* Higher Diploma (master degree or higher)

In total, 16 semi-structured interviews were conducted with participants from different organizational levels. To ensure participant anonymity, interviews were sequentially numbered as they occurred using an acronym based on roles in the implementation process; TL; top leader, MM; mid-manager, and RN; registered nurse.

### Observations

In both wards, observations were carried out by the PI. The observations can be helpful in documenting current processes [36] as well as assessing local contexts and observing the nature and intensity of how interventions are being implemented [37]. Before commencement, the PI was introduced to staff to reiterate research objectives. This ensured that the PI was accepted in both teams and was not a stressor for shadowed staff. Staff were therefore comfortable with their actions, facilitating “real-life” observations of daily workflows. Observations were conducted over different days, ensuring at least one full shift in each ward was conducted. To each nurse, the PI explained the purpose of the observations, which was to identify and not judge their daily practices. In France and across the nursing profession, trainees typically shadow nurses, therefore the PI directly integrated into the staff dynamic. This factor with the observation duration limited the “Hawthorne effect” or observation bias [38]. As a registered nurse, the PI comprehended the different actions and became familiar with the work environment. To prevent over familiarity and retain a critical distance, only descriptive non-judgmental notes were taken [39].

### Document collection

Various documents were collected from both wards, e.g., patient file documentation, traceability records such as checklists, blood transfusion follow-up, hemodialysis follow-up, working procedures and policies, and “Bord” table as indicators for staff performance. The PI was also introduced to the hospital informatics system (Dx Care) and was permitted to review electronic forms. During observations period, the PI also attended staff and quality meetings.

### Research ethics

In France, research involving human in three types of study: interventional studies, studies with minimal risk and intervention, and non-interventional studies (in the usual framework of patient), requires an ethical approval from an ethical committee, the “Jarde law” L1121-1 PHC ( LOI n° 2012 − 300 du 5 mars 2012) [40]. This study involved only professionals and the content of activity, without patient involvement or human experiments, it does not require an IRB clearance in the way it is understood in the United States [41, 42]. It requires only an administrative approval and this was gained through convention before data collection and interviews was signed between the French School of Public Health and the teaching hospital. This convention defined the study duration and the investigations to be carried out.

The study was conducted in accordance with ethics in qualitative research guidelines [43]. A signed consent form was obtained from interviewees to formalize their willingness to participate. The PI was highly sensitive to confidentiality issues and conducted interviews in private offices in comfortable and informal settings. However, some Med Rea nurse interviews were conducted at nursing stations which facilitated rapid access to critical patients. All interviewees and interview transcripts were anonymized and assigned acronyms.

### Data storage

Interviews transcripts were stored in two different Excel sheets; one devoted to leaders and managers and one for nurses. Sheets were divided into questions, and each column represented one interviewee. Answers were accorded to the related question, thereby maintaining one concept in each row/column ‘case’. All datasets were stored on an encrypted access computer which required a password.

### Data analysis

Analyses were conducted by the PI. The first stage involved a rigorous inductive analysis of interview transcripts [44]. Narratives reflecting certification procedures and implementation processes were extracted and organized according to type (e.g., action, interaction, actors, key component facilitators, barriers, context preparedness, and others). These narratives were then used in the second step and deductively analyzed using pre-identified conceptual frameworks [45]; the Quality Implementation Tool (QIT) and the Translational Mobilization Theory (TMT). The interpretation of observations and document reviews were both used as support datasets. In the observations, we followed how certification procedure practices were embedded in the daily practices, and analyzed how they were effectively integrated. In relation to documents, we went through each wards’ action plan for certification implementation, reviewed supportive documents such as policies and working procedure, and assessed their usefulness for successful implementation. Each data analysis stage was reviewed and discussed with the second author to ensure analysis credibility (Additional file 3 shows a study checklist using the Consolidated Criteria for Reporting Qualitative Research (COREQ) checklist).

The second analysis stage was two-fold: the first approach investigated the implementation of certification procedures using a generic implementation tool, i.e., QIT. This is a user-friendly pragmatic tool developed based on an exhaustive review of literature summarizing 25 implementation frameworks, regardless of the intervention, environment, or results [19]. The QIT encompasses six major components; (1) develop an implementation team, (2) foster a supportive organizational/climate and conditions, (3) develop an implementation plan, (4) Receive training and technical assistance, (5) practitioner-expert collaboration, and (6) evaluate the effectiveness of the implementation. These components presented in a tabular format, with each component divided into action steps in each row, and each row divided into three columns. These columns represented three distinct steps over the implementation process, i.e., (i) planning, (ii) real-time monitoring, and (iii) innovation evaluation. QIT was primarily developed to implement innovation with quality [46]. In this study, QIT constructs were used to frame emergent themes from interview transcript analyses. This was conducted by aligning tool components with actions and themes derived from manager and leader interviews (Additional file 1).

This first approach was generic in nature; the QIT allowed the capture of transversal elements involved in the implementation of quality procedures. However, we lacked an integrated approach to these factors in the local socio-material context. The consideration of socio-material contexts allows for a better understanding of interactions between the local context of implementation and the development of various factors [27], e.g., the implementation of informatics tools and leadership depends on local work dynamics. These elements were the core of the second approach, or TMT.

TMT is based on ethnographic research on organizing the work of nurses involved in patient care pathways [47]. Nurses are “obligatory passage points” in hospitals which localize, refract, and shape materials and activities supporting patient care pathways [48]. This systematic framework allows researchers to capture emerging contextually complex procedures during service processes [49]. TMT embraces social, material, and cognitive processes, leading to practice fulfilment. TMT core components comprise: ‘project’ which is a goal-oriented strategic activity mobilized through ‘mechanisms of mobilization’ (Table 2), across a ‘strategic action field’. This latter term is defined by resources and conditions which enable and shape project mobilization [47, 49]. TMT was previously implemented in several different case studies, healthcare trajectory and multidisciplinary research projects [50, 51]. TMT was also used to analyze the local context of nurse activities and explore the emergence of certification processes which were defined as “collaborative work practices” [28] in daily workflows. In this study TMT components were helpful in capturing local socio-material factors emerging from interviews analyses and observations, e.g., interactions between actors and innovation. As a result, we identified interaction mechanisms within the framework. This was based on triangulation between managers, nurse interviews, and shadowed observations, all of which were aligned to TMT core components (Additional file 2).

**Table 2:** Mechanisms of Mobilization of TMT[49]

Mechanisms of Mobilization	Definition
Object formation	“practices that create the objects of knowledge and practice and enroll them into a project”
Work articulation	“practices that assemble and align the elements (people, knowledge, materials, technologies, bodies) through which object trajectories are mobilized within projects”
Translation	“practices that enable practice objects to be shared and differing viewpoints, local contingencies, and multiple interests to be accommodated in order to enable concerted action”
Reflexive monitoring	“practices through which actors evaluate a field of action to generate situational awareness of project trajectories”
Sense-making	“practices though which actors interpret, order, construct and account for projects and at the same time produce and reproduce institutions”

## Results

In addition to interviews, 83 observational hours were also conducted over four separate weeks. These were divided as follows; one module in the Med Rea ward over 40 h, and two interventional rooms in the Dig Endo ward over 43 h. All nurses were interviewed and observed on their daily shift. We therefore obtained a comprehensive description of all tasks in a complete working shift in both wards. This allowed the PI to focus on how nurses interacted with tasks related to certification procedures, e.g., patient file documentation, checklists, medication administration, and others.

The following sections outline the data retrieved in this study; part I shows emerging elements from certification implementation using QIT. Part II localizes these components within the activity’s context, with different mobilization mechanisms.

### Part I

Our results showed that the majority of elements were captured by the QIT components and action steps, further details in (Additional file 1). Results showed that the “implementation team” in charge of certification implementation were well developed and structured, as mentioned by interviewees. The implementation team consisted of a process leader who managed the implementation process at an institutional level. They could be a physician or an MM working with: executive managers, the experts in field such as hygienist for infection control procedures, and professionals (nurse or caregivers), the referents, the quality engineer and a steering committee e.g., the committee for nosocomial infection prevention. All worked in collaboration with the TL.

The second component, “Foster a supportive organizational climate and conditions”, identified several key essential elements for the successful implementation at professional level, such as a key actor with a ‘referent of action’ role. Referents are professionals who assist new implementation processes “*for example there is a nurse referent for hygiene; she disseminates new procedures and best practices to teams”* TL_1_. Other elements included the communication of procedural needs and benefits, and the professional implication of such implementation. These were considered helpful actions in avoiding professional resistance to intended changes. Other actions enhanced accountability by using a quality management system (QMS), conducting a pilot study prior to implementation and effective communications and shared decision-making processes. In addition to the presence of an administrative support for the implemented intervention such as working procedures, protocols etc… either in paper or electronic forms.

The “receive knowledge and/or technical assistance” construct was identified by managers; *“Before implementation we defined what training was needed for professionals and the required technical support…”* MM_1_.

 Certification implementation occurred according to a program and an action plan defined for each department and ward. This was developed based on national recommendations as identified by the HAS certification manual, and each sectors’ CQ. This latter step reflected the identified risks in priori and posteriori for each sector and it was considered a roadmap for risk management. This program defined a set of tasks corresponding to each standard objective over predefined timelines (The Dig Endo action plan) and responded to the “Develop an implementation plan” component.

The fifth component; “Practitioner-developer collaboration” was not applicable to certification implementation procedures, whether there is no innovation developer, and hospitals implemented procedures developed based on the national recommendations. These recommendations are defined in the HAS certification manual and each hospital develop their action plan accordingly to these recommendations. For the “Evaluate the effectiveness of the implementation” component, interviewees identified quantitative and qualitative evaluation strategies which were carried out differently, according to the intended action. It was based on the evaluation leaders of change readjust and adapted intervention to improve implementation effectiveness, “ *it was the ability to conduct a pre-test (for the intended change), an auto-evaluation procedure and receiving feedbacks from each sector thereby allowing us to see what we could do to improve because the auto-evaluation allowed us to identify missing elements”* TL_1_.

In addition to these comments, TL also cited major barriers to the implementation of certification in different wards at the hospital, and cited a lack of organizational support, time, information, human resources, a generalized professional resistance, and an overall challenging process.

### Part II

This part of the study framed the identified components at the activity level. It entails previous result analyses by explaining the different inter-relationships at the local context.

The HAS identified healthcare system priorities, and each subject under these priorities included a set of standards and indicators [52]. These standards underpinned the quality program of each healthcare facility as well as the policies and objectives of the QMS [41]. Hence, the higher goal of the healthcare system - defined by care quality and patient safety - represented ‘organizing logic’ which determined the scope of possible actions and activities within facilities, and shaped its purpose. The primary mobilization of certification procedures initiated within departments was based on a list of priority actions previously elaborated through the CQ. This occurred via a set of actions steps according to each sector action plan “we *have an action plan and a list of priority actions, and annually, we contact the quality engineer to revise this action plan”* MM_1_. Interventions leading to the emergence of certification in the ward were introduced to nurses by mid-managers and/or by the referent, and this process reflected the ‘object formation’ mechanism. Interventions may took the form of new technologies and/or materials supporting practices, or interpretative repertoires such as protocol changes, policies, checklists and/or traceability documents. Through these interventions, nurses translated recommendations and certification criteria or other quality policies into practice. For example, in the Dig Endo ward nurses were using a working protocol to support preparations for the pre- and on-going of new adopted change of intervention. The change leader - who led the implementation at the professional level - disseminated the information on the required changes to nurses, its needs and benefits in terms of patient care. In other words, the message was how change meet the facility’s organizational logic, thereby reflecting a ‘translation mechanism’. This was seen in nurse interviews; they perceived the importance and the need of certification procedures to improve patient care quality *“certification procedures are progress and enhancement tools which improve patient care”* RN_4_.

Healthcare systems by their very nature are dynamic with changeable actions; thus, monitoring processes is important, particularly when implementing cross-sector processes or actions. In order to ensure work harmonization between different sectors. For example, in the Dig Endo ward, the implementation of a checklist was intended for ‘with and without’ general anesthesia (GA) units. The checklist was successfully implemented at the ‘with’ GA unit, but it was not successful in the ‘without’ GA unit. According to MM_1_, the checklist was developed as a coordination sheet between the doctor and anesthetist; however, in the ‘without’ GA unit, there was no anesthetist, but only a coordination between doctors and nurses which generated a lack of monitoring data. This information was used by the change leader, who worked with other departments on a new checklist applicable to the Dig Endo ward and other interventional wards, such as interventional radiology. Changes were re-implemented and monitored to assess workability and acceptability among nurses. This ‘work articulation’ between multi-levels and sectors was fundamental for the successful integration of implemented checklist. It occurred at team and departmental meetings, alongside the on-going monitoring of integrated changes.

The evaluation of the implementation occurred continuously throughout the process, both formally and informally. This was done to describe the occurrence and positioning of the implemented intervention at the activity’s level, as well as from the organization’s perspective, indicating a ‘reflexive monitoring’ mechanism; *“We have monthly performance tables*…*we have follow-up indicator tables that we monitor monthly or once every semester or annually, and we also have morbidity rates which are monitored every two months*” MM_2_. Whenever there was a drop in indicators or an adverse event, analyses occurred and corrective actions taken. For example *“one day there was a big alert, endoscopes were contaminated and we looked for possible causes. We did not understand because all staff were well trained. After analyzing the situation, we realized instruments were overbooked; nurses and caregivers were under pressure and were reducing decontamination steps for the endoscopes. So we developed organization tables and we make sure doctors organized between them and avoid these overbooking. This information was passed on during our team meeting”* MM2. Another example from the Med Rea ward involved nurses who were using new intubation systems by tracing extubation rates, and were relaying their negative experiences at meetings. This feedback was considered a primary support in evaluating change feasibility and outcomes for patient care. Thus, nurses and managers were keen to improve, “*we reverted to our action plan and adjusted according to adverse events”* MM_1_. The mobilization of intervention at the nurse level also depended on a ‘sense-making’ mechanism. In the nursing field, nurses are actively engaged with certification procedures, e.g., they are involved in protocol preparation and validation, they provide and share experiences, and they contribute to auditing systems. By involving nurses in the implementation process, actions and/or care processes evolve into their practices, meaning this active engagement is invaluable for a successful change implementation in the activity system. Professional active engagement provides meaning and allows appropriate team-based action mechanisms.

Finally, leaders emphasized the role of MM and their ability to conduct a participative strategy over the implementation process in order reach a successful integration “*an implementation depends on the mid-managers, and what they disseminate between departments. But, each department has its own reality and the ability of each mid-manager to conduct an implementation effectively”* TL_2_.

**Fig. 2:**
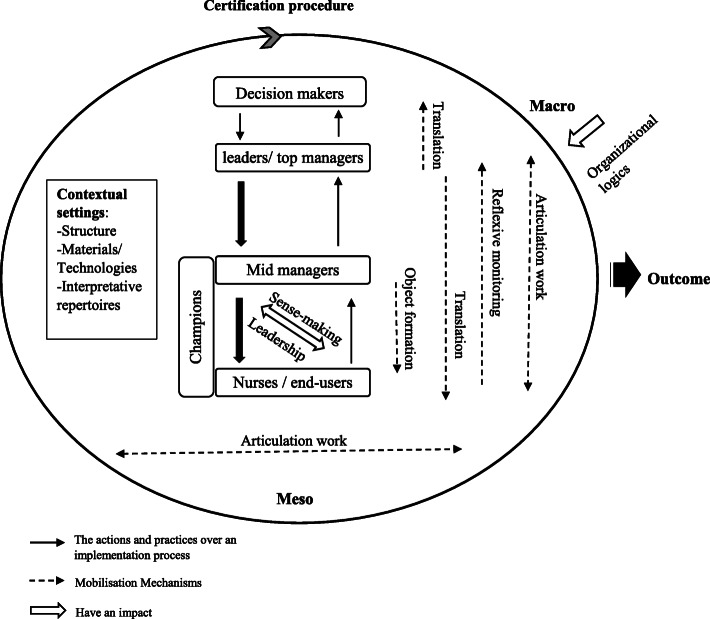
The proposed framework

Both parts guided the construction of proposed framework (Fig. 2) by understanding how the implementation process of certification procedures occurs through key elements and mechanisms of mobilization shaping the interrelationships between actions, actors, and the local context.

Our framework (Fig. 2) presents both levels incorporated into the implementation process; Macro and Meso levels. The Macro reflects healthcare systems by the organizational logics and the Meso reflects the organizational level which comprises the following core components; contextual settings: structure, materials, technologies, and interpretative repertoires. The actors implicated in certification implementation procedures are from different organizational levels. Champions are represented beside mid-managers and nurses levels because they emerge from both levels. The leadership approach and mobilization mechanisms shape interrelationships between the framework components including object formation, translation, work articulation, reflexive monitoring, and sense-making. Solid arrow thickness reflects the importance of the implementation strategy type (top-down or bottom-up). The iterative aspect of certification is represented by the circle shape and the arrow which reflects the continuity of this procedure. Finally, the outcome reflects the quality and safety of care provisions.

## Discussion

In this case-study, we expanded the understanding on the quality policy implementation in the activity system by developing an implementation framework for certification procedures in hospitals, at nurse level. The framework was constructed using a two- step mixed approach. In the first stage, the inductive analysis led to the identification of key elements for the certification procedures implementation. In the second, these elements were analyzed using two theoretical approaches the QIT and the TMT. QIT helps to capture the following framework components. First, the team in charge of certification procedures were characterized by a position and tenure diversity, which is considered essential criteria for a well-balanced and effective implementation team [53]. Although team members were changing depending on the implemented procedure, stability was always maintained in the roles. Second, elements related to a favorable organizational climate conditions, such as contextual settings, knowledge, resources, and material availability are fundamental for certification integration [54, 55]. The administrative supports, such as policies and operational protocols are major facilitators of professional practice, in terms of actions and/or processes [56]. The lack of any of these factors in addition to time, may constitute –according to interviewees- a major constraint hindering implementation of the desired change [57]. Pettigrew et al., presents multiple contextual factors contribute to a strategic change [58]. Typically, a supportive organizational culture and individuals leading the change are locally instrumental for the integration process [59]. In line with this, our study showed that over the certification implementation process at the local level, the ‘referent of action’ played an essential role and it appeared they adopted the champion role. Champions may emerge during an implementation process, sometimes as part of an intervention, sometimes as part of an implementation strategy, and at other times not at all, i.e., they thrive in the implementation environment [60]. They act as mediators between nurses and managers with a capacity to disseminate information and support mobilized actions [61]. These champions - who are sometimes nurses - deployed, followed, monitored, and reflected peer experiences to improve change acceptability and sustainability. Due to their familiarity with the context, they identified the required contextual elements and local context readiness to deploy the desired changes [62]. Thus, champions are key performers in the certification implementation process [63].

Another elements identified at the local level was the leadership approach [64] of proximity managers or the change leader [65]. It allows an active engagement of nurses through a participative strategy used over the implementation processes [66, 67]. In parallel, came the “sense-making” mechanism identified by the TMT. The possibility to experiencing a change feasibility by nurses and providing feedback on its organizational fit support the acceptability of implemented intervention in their practices [68, 69] and avoids resource wastage [70]. Change leaders and nurses must determine the pace and extent of change implementation and its feasibility within their service [71]. A ‘supportive leadership’ approach used by the implementer [71] and a ‘sense-making’ mechanism both determine how professionals translate change into practice, to meet desired outcomes [49]. Additionally, local managerial support of the implemented intervention was essential [72]. This emerges by communicating the needs and benefits of certification procedures with nurses and decision makers [56, 66] under “translation” mechanisms [48]. Understanding the meaning and importance of change is an important precondition for successful implementation. This comes from the notion that nurses may perceive the implemented intervention as a threat affecting their routines, and thus they resist the change [73]. In addition, the identified actions under the “work articulation” mechanism [49], such as continuous communication between managers, and sectors over the implementation process was essential, It helps settle issues in confrontational situations [56]. These key junctures relied a well on a shared culture and staff learning; they formalizing workflow trajectories and ensuring work harmonization and staff commitment, thus achieving effective implementation [74]. An on-going evaluation all over the implementation process, comes under a “reflexive monitoring mechanisms”, was considered essential element. Champions and nurses feedbacks, as well as, formal evaluation systems such as auditing help monitoring the position of implemented intervention, enhance and adjust the process toward reach the desired outcomes [65, 66].

Our research contributes to and extends understanding and knowledge on “how” and “what” influences the implementation of these quality policies in nurses’ work. The dynamic aspect of contextual factors may impede implementation in one setting and facilitate it in another [75]. Knowing these factors [76] and how they interrelate during an implementation process is essential towards an effective implementation at the activity level [77]. This framework goes beyond the typical perspective of a conventional framework [18] as it considers local context mechanisms which shape and guide an implementation process, this was facilitated using TMT components [49]. The framework shows how key attributes and elements from local contexts interacted via multiple mobilization mechanisms, reflecting the impact of local socio-material contexts [29]. An organization’s life occurs throughout an ‘entanglement’ between the materials and the social context and the way the actor and artefacts ‘entail each other in practice’ [78]. Characterizing and exploring the key elements and the socio-material context of an implementation allows implementers to consider a broader vision on what influences a successful implementation outcome. In line with this, our suggested framework characterizes certification implementation in a hospital. We presented how an implementation context is composed from both social and material elements, which interact together in a continuum rather than in a linear “pipeline” manner [79].

## Study limitations

Our study had several limitations. Firstly, in the interview guide, we included no direct questions which developed the different QIT components, but elements were retrieved from interviewee narratives and matched by the different action steps. This may explain the absence of some action steps from the analysis table. Secondly, some data may have been missed from nurse interviews due to extenuating circumstances; nurses had to interrupt interviews to check and respond to patients. This elicited brief responses and may not have adequately reflected their opinion. Thirdly, nurses were not observed and followed over long periods for certification preparation. Observations were conducted to determine the emergence of certification practices in daily workflows, and to investigate work organization and coordination between proximity managers and nurses. Finally, because this was an exploratory study in one setting, our data cannot be extrapolated to all hospital settings.

## Conclusions

We propose a framework which analyses and describes the implementation of certification procedures at nurse level. Our observations were generated using two different approaches; practical implementation science using QIT, and the TMT approach which is a sociological model derived from implementation science perspectives. TMT was highly beneficial in understanding the emergence of certification within the local context of nurse activities. It allowed us to identify interactions between nurses, managers, the implemented intervention, and the context. It went beyond the systematic framework, to the actual reality of activity system complexity. In the future, we will test this framework in national and international empirical studies.

## Supplementary Information

**Additional file 1:** Table analysis using the quality implementation tool (QIT).

**Additional file 2:** Table analysis using translational mobilization theory (TMT).

**Additional file 3:** Study reporting using the COREQ checklist.

## Data Availability

Datasets (which include individual transcripts) are not publicly available due to confidentiality policies. However, they may be obtained from the corresponding author upon reasonable request.

## Abbreviations

HAS: Haute Autorité de Sante (French National Health Authority)
ISO: International Organization for Standardization
IQSS: Indicateur de Qualité et Sécurité des Soins (Healthcare quality indicators)
CQ: Compte qualité (quality account monitoring tool)
EHR: Electronic Health Record
IDE: Infirmiers Diplômé d’Etat (Registered nurse qualification)
QMS: Quality management system
GA: General anesthesia

## Declarations

### Authors' information

IS is a doctoral student at the School of Public Health EHESP, Rennes, France.

MW is associate professor at MOS-EHESP, Paris/Rennes, France and lecturer at the Global Health Institute, Geneva University, Geneva, Switzerland.
