# Supplementary material for: A framework for the implementation of certification procedures in nurse level: a mixed approach study
Source: BMC Health Serv Res. 2021 Sep 8;21:932. doi: 10.1186/s12913-021-06940-0 (PMC8425162; doi:10.1186/s12913-021-06940-0)
Supplement: Supplementary file 1 — Additional file 1: Table analysis using the quality implementation tool (QIT). [file 12913_2021_6940_MOESM1_ESM.docx]

**A framework for the implementation of certification procedures at nurse level: a case study in a French hospital.**

*Israa SALMA¹*, Mathias WAELLI^2,3^*

^1^École des Hautes Etudes en Santé Publique, EA 7348 MOS,  Rennes, France. E-mail: [israa.salma@eleve.ehesp.fr](mailto:israa.salma@eleve.ehesp.fr)

^2^École des Hautes Etudes en Santé Publique, EA 7348 MOS, Paris, France. E-mail : [mathias.waelli@ehesp.fr](mailto:mathias.waelli@ehesp.fr)

^3^Global Health Institute, Geneva University

*corresponding author

**Table 1: results of analysis using the QIT (1)**

| **Components** | **Action steps** | **Total: 8** |
| --- | --- | --- |
| **1. Develop an implementation team** | 1.1 Decide on structure of team overseeing implementation | 6 |
|  | 1.2 Identify an implementation team leader | 8 |
|  | 1.3 Identify and recruit content area specialists as team members | 8 |
|  | 1.4 Identify and recruit other agencies and/or community members such as family members, youth …as team members | 4 |
|  | 1.5 Assign team members roles, processes, and responsibilities | 7 |
| **2. Foster supportive organizational/**  **communitywide climate and conditions** | 2.1 Identify and foster a relationship with a champion for the innovation | 6 |
|  | 2.2 Communicate the perceived need for the innovation within the organization | 8 |
|  | 2.3 Communicate the perceived benefit of the innovation within the organization | 8 |
|  | 2.4 Establish practices that counterbalance stakeholder resistance to change | 8 |
|  | 2.5 Create policies that enhance accountability | 8 |
|  | 2.6 Create policies that foster shared decision-making and effective communication | 8 |
|  | 2.7 Ensure that the program has adequate administrative support | 8 |
| **3. Develop an implementation plan** | 3.1 List tasks required for implementation | 7 |
|  | 3.2 Establish a timeline for implementation tasks | 6 |
|  | 3.3 Assign implementation tasks to specific stakeholders | 8 |
| **4. Receive training and technical assistance** | 4.1 Determine specific needs for training and/or TA | 4 |
|  | 4.2 Identify and foster relationship with a trainer(s) and/or TA provider(s) | 4 |
|  | 4.3 Ensure that trainer(s) and/or TA provider(s) have sufficient knowledge about the organization/community’s needs and resources | NM |
|  | 4.4 Ensure that trainer(s) and/or TA provider(s) have sufficient knowledge about the organization/community’s goals and objectives | NM |
|  | 4.5 Work with TA providers to implement the innovation | 3 |
| **5.Practitioner–**  **expert collaboration** | 5.1 Collaborate with expert about factors impacting quality of implementation in the organization | NA |
|  | 5.2 Engage in problem solving | NA |
| **6. Evaluate the effectiveness of the implementation** | 6.1 Measure fidelity of implementation (i.e., adherence, integrity) | 5 |
|  | 6.2 Measure dosage of the innovation | 4 |
|  | 6.3 Measure quality of the innovation’s delivery qualitative aspects of program delivery (e.g., implementer enthusiasm..) | 5 |
|  | 6.4 Measure participant responsiveness to the implementation process | 5 |
|  | 6.5 Measure degree of program differentiation | 8 |
|  | 6.6 Measure program reach | 8 |
|  | 6.7 Document all adaptations that are made to the innovation | 7 |

**Table 1:** The methodology of analysis according to the Quality Implementation Tool (QIT) was conducted by identifying the presence or absence of each components and their action steps in interviewees’ narratives, mainly managers and top leaders. This is in terms of the implementation strategy of certification procedure and the key elements for successful implementation. The following coding was used in the analysis: present/ yes: **1**; not present/No: **0**; Not mentioned by interviewees: **NM**; and not applicable to studied procedures: **NA**. As final step an overall of ‘yes’ is presented in the following table for each action step.

**References**

1. Meyers DC, Katz J, Chien V, Wandersman A, Scaccia JP, Wright A. Practical implementation science: developing and piloting the quality implementation tool. Am J Community Psychol. 2012;50(3–4):481–96.
