# Supplementary material for: A framework for the implementation of certification procedures in nurse level: a mixed approach study
Source: BMC Health Serv Res. 2021 Sep 8;21:932. doi: 10.1186/s12913-021-06940-0 (PMC8425162; doi:10.1186/s12913-021-06940-0)
Supplement: Supplementary file 2 — Additional file 2: Table analysis using translational mobilization theory (TMT). [file 12913_2021_6940_MOESM2_ESM.docx]

**A framework for the implementation of certification procedures at nurse level: a case study in a French hospital.**

*Israa SALMA¹*, Mathias WAELLI^2,3^*

^1^École des Hautes Etudes en Santé Publique, EA 7348 MOS,  Rennes, France. E-mail: [israa.salma@eleve.ehesp.fr](mailto:israa.salma@eleve.ehesp.fr)

^2^École des Hautes Etudes en Santé Publique, EA 7348 MOS, Paris, France. E-mail : [mathias.waelli@ehesp.fr](mailto:mathias.waelli@ehesp.fr)

^3^Global Health Institute, Geneva University

*corresponding author

**Table 2: Results of analysis for certification implementation using TMT (1)**

| **Components** | **Elements** | **“how of” within the studied hospital field** |
| --- | --- | --- |
| **Projects**  **Institutionally sanctioned strategic activity** | **Project** | Certification procedure |
|  | **Sub project** | Example: certification within digestive endoscopy sector |
|  | **Project actor** | Quality management system / quality programme |
|  | **Intersecting project** | Direct patient care, organisational work |
| **Strategic action field**  **(teaching hospital centre)** | **Organizing logics** | The care quality and patient safety |
|  | **Structures** | Different hospital departments, example digestive endoscopy sectors, care quality direction and health manager, executive manager, quality engineers, endoscopy steering commission, local managers, processes pilots, multidisciplinary professionals (doctors , nurses, care providers.. ), technicians |
|  | **Materials/technologies** | Infrastructure of digestive endoscopy (with and without general anaesthesia, emergency), different machineries and caregivers’ tools of patient care (new endoscope…). Patient electronic health records, informatics system |
|  | **Interpretative repertoires** | Protocols and policies, procedures, meeting reports, action plan; documentation system: checklist HAS endoscopy; ecology paper, patient file; traceability endoscopy (paper and electronic format) |
| **Mechanisms of mobilization** | **Object formation** | Introduce the intended action, materials and/or interpretative repertoires supportive for the emergence of certification in wards, by the proximity managers or referents of actions to professional (mainly nurses), this is according to each sector action plan defined on the action priority by the top and proximity managers and professionals. |
|  | **Articulation work** | Support and accompaniment of action, actions’ referents roles (professionals), ensure regular and /or necessary formation and technical assistance for professionals, the presence of shared culture, the regular and on-going meetings over the process of implementation, between the referent of action and proximity manager, the steering committee and managers, departmental meetings especially for the cross-sectional care process or practices, quality meeting in each department. Multidisciplinary team work, nurse organizing work and coordinating with other professional. |
|  | **Translation** | The proximity managers and action referents communicate with nurses the perceived benefits and needs of the implemented action and certification procedure, as well as, top leaders ensure to diffuse the importance of the intended change over a certification between the different managers and decision makers. And present how it can meet the organisation vision and mission, in terms care quality and safety improvement and also for the public authority. |
|  | **Reflexive monitoring** | nurses’ feedbacks during team meetings, and from the referents of action about the action feasibility and acceptability, the presence auditing system, the follow up of performance tables, Bord table, adverse events rate, morbidity, different indicators allied with the objective of action plan |
|  | **Sense-making** | The nurses’ active engagement in the process of implementation, and nurses play the role of referent of action, nurses are implicated to redact and up-date protocols, nurses take the responsibility to carry audit in other departments, there are test phase for the action nurses responsible to give their feedback on the change implementation and how to improve to gain it sustainability. |

**Table 2**. presents the results of analysis using the Translation Mobilisation Theory (TMT) (1). The methodology of analysis was conducted based on Operationalizing TMT table (2), the interviewees’ narratives and supported by the observations and collected documents. The following table presents the core components of TMT which are: the project, the strategic action fields and the mechanisms of mobilizations, and their elements, and in third column how it was figured out from the experience of certification implementation within studied teaching hospital centre.

**Reference**

1. Allen D, May C. Organizing Practice and Practicing Organization: An Outline of Translational Mobilization Theory. SAGE Open. 2017 ;7(2):2158244017707993.
2. Davina Allen. Development [Internet]. Translational mobilisation theory. 2018 [cited 2020 Mar 20]. (Available from https://www.translationalmobilisationtheory.org/using-tmt)
