# Supplementary material for: A framework for the implementation of certification procedures in nurse level: a mixed approach study
Source: BMC Health Serv Res. 2021 Sep 8;21:932. doi: 10.1186/s12913-021-06940-0 (PMC8425162; doi:10.1186/s12913-021-06940-0)
Supplement: Supplementary file 3 — Additional file 3: Study reporting using the COREQ checklist. [file 12913_2021_6940_MOESM3_ESM.docx]

**A framework for the implementation of certification procedures at nurse level: a case study in a French hospital.**

*Israa SALMA¹*, Mathias WAELLI^2,3^*

^1^École des Hautes Etudes en Santé Publique, EA 7348 MOS,  Rennes, France. E-mail: [israa.salma@eleve.ehesp.fr](mailto:israa.salma@eleve.ehesp.fr)

^2^École des Hautes Etudes en Santé Publique, EA 7348 MOS, Paris, France. E-mail : [mathias.waelli@ehesp.fr](mailto:mathias.waelli@ehesp.fr)

^3^Global Health Institute, Geneva University

*corresponding author

**Table 3: Study reporting using COREQ checklist (1)**

| **No .** | **Item .** | **Guide questions/description .** | **Study reporting** |
| --- | --- | --- | --- |
| **Domain 1: Research team and reflexivity** |  |  |  |
| Personal Characteristics |  |  |  |
| 1. | Interviewer/facilitator | Which author/s conducted the interview or focus group? | The corresponding author |
| 2. | Credentials | What were the researcher's credentials? *E.g. PhD, MD* | Registered Nurse “RN” and PhD student |
| 3. | Occupation | What was their occupation at the time of the study? | Third year PhD student |
| 4. | Gender | Was the researcher male or female? | Female |
| 5. | Experience and training | What experience or training did the researcher have? | Experience in nursing profession, healthcare system and quality management. Training on methods and analysis of qualitative data; qualitative research methodology; articles redaction. |
| Relationship with participants |  |  |  |
| 6. | Relationship established | Was a relationship established prior to study commencement? | There is no relationship established prior to study |
| 7. | Participant knowledge of the interviewer | What did the participants know about the researcher? e*.g. personal goals, reasons for doing the research* | Participants only know a general intention of the study which is understand the implementation process of certification procedures in the hospital.  . |
| 8. | Interviewer characteristics | What characteristics were reported about the interviewer/facilitator? e.g. *Bias, assumptions, reasons and interests in the research topic* | The interviewees were carried by the corresponding author. Nurses’ interviews were conducted in their working shift this may lead to shorter and incomplete answers due to their limited availabilities |
| **Domain 2: study design** |  |  |  |
| Theoretical framework |  |  |  |
| 9. | Methodological orientation and Theory | What methodological orientation was stated to underpin the study? *e.g. grounded theory, discourse analysis, ethnography, phenomenology, content analysis* | Study used an mixed approaches, inductive and deductive approaches  The methodological orientation was according to content analysis using two theoretical approaches, the practical implementation sciences and a middle range theory  Analysis were integrated and translated in an implementation framework |
| Participant selection |  |  |  |
| 10. | Sampling | How were participants selected? *e.g. purposive, convenience, consecutive, snowball* | Participants were selected in a purposive way, in the first step they have to be involved in the process of implementation of certification, in the second step only nurses were interviewed because we interested to study the nurse role are engaged in the process of implementation in their daily workflow. All interviews were carried after confirmation to participate in the study |
| 11. | Method of approach | How were participants approached? e*.g. face-to-face, telephone, mail, email* | Face to face semi structured interviews, |
| 12. | Sample size | How many participants were in the study? | Sixteen interviews |
| 13. | Non-participation | How many people refused to participate or dropped out? Reasons? | No one |
| Setting |  |  |  |
| 14. | Setting of data collection | Where was the data collected? e*.g. home, clinic, workplace* | In the workplace at hospital |
| 15. | Presence of non-participants | Was anyone else present besides the participants and researchers? | Participants were interviewed alone and in a private place generally |
| 16. | Description of sample | What are the important characteristics of the sample? *e.g. demographic data, date* | For nurses the average of experience is 9 years in theirs services and 43 years old. All have same qualification level of studies “RN” |
| Data collection |  |  |  |
| 17. | Interview guide | Were questions, prompts, guides provided by the authors? Was it pilot tested? | Interviews were conducted according to a semi structured interview guide. Tested on nurses out of the studied hospital |
| 18. | Repeat interviews | Were repeat interviews carried out? If yes, how many? | No |
| 19. | Audio/visual recording | Did the research use audio or visual recording to collect the data? | Yes, all interviews were recorded |
| 20. | Field notes | Were field notes made during and/or after the interview or focus group? | After each interview, a brief notes were registered for the important emergent ideas |
| 21. | Duration | What was the duration of the interviews or focus group? | Leaders and managers: 30-45 min  Nurses: 20-30 |
| 22. | Data saturation | Was data saturation discussed? | Yes, interviews have been stopped, when their output reach a closing sense |
| 23. | Transcripts returned | Were transcripts returned to participants for comment and/or correction? | No |
| **Domain 3: analysis and findings** |  |  |  |
| Data analysis |  |  |  |
| 24. | Number of data coders | How many data coders coded the data? | coding data were used in limited part of the study,  and was conducted manually by the corresponding author and revised by the co-author |
| 25. | Description of the coding tree | Did authors provide a description of the coding tree? | Yes the used code were described briefly in legend of table |
| 26. | Derivation of themes | Were themes identified in advance or derived from the data? | themes were derived from the analysis of interviews |
| 27. | Software | What software, if applicable, was used to manage the data? | Data were analysed manually on excel sheets |
| 28. | Participant checking | Did participants provide feedback on the findings? | No |
| Reporting |  |  |  |
| 29. | Quotations presented | Were participant quotations presented to illustrate the themes / findings? Was each quotation identified? e*.g. participant number* | Yes, some of the participants quotations are presented in the manuscript to support the themes and findings, and it identified according to the professional respondent according to a defined acronym and a sequence number |
| 30. | Data and findings consistent | Was there consistency between the data presented and the findings? | Yes there was a consistency between the emergent themes, the used tools and the findings are translated in an implementation framework |
| 31. | Clarity of major themes | Were major themes clearly presented in the findings? | In the discussion part of the study while presenting the developed framework |
| 32. | Clarity of minor themes | Is there a description of diverse cases or discussion of minor themes? | Not applicable |

**Table 3** presents the study report using the “Consolidated criteria for reporting qualitative research” COREQ checklist (1), we added a new column to the 32 item checklist and in which we answered about our study, the guide questions which described each item from the checklist.

1. Allison Tong, Peter Sainsbury, Jonathan Craig. Consolidated criteria for reporting qualitative research (COREQ): a 32-item checklist for interviews and focus groups, *International Journal for Quality in Health Care*, Volume 19, Issue 6, December 2007, Pages 349–357
